# First Molecular Identification of *Babesia*, *Theileria*, and *Anaplasma* in Goats from the Philippines

**DOI:** 10.3390/pathogens11101109

**Published:** 2022-09-27

**Authors:** Eloiza May Galon, Rochelle Haidee Ybañez, Adrian Miki Macalanda, Giemelene Rose Estabillo, Margaret Therese Rose Montano, Marielle Danise Veedor, Anatolio Garvida, Ralph Joselle Fabon, Mary Ruth Callanta, Kim Joseph Labutong, Maria Agnes Tumwebaze, Benedicto Byamukama, Shengwei Ji, Iqra Zafar, Adrian Ybañez, Xuenan Xuan

**Affiliations:** 1 National Research Center for Protozoan Diseases, Obihiro University of Agriculture and Veterinary Medicine, Obihiro 080-8555, Japan; 2 Institute of Molecular Parasitology and Protozoan Diseases, Main Campus and College of Veterinary Medicine, Barili Campus, Cebu Technological University, Cebu City 6000, Philippines; 3 College of Science, University of the Philippines Cebu, Cebu City 6000, Philippines; 4 Department of Immunopathology and Microbiology, College of Veterinary Medicine and Biomedical Sciences, Cavite State University, Indang 4122, Philippines; 5 Saddle & Clubs Leisure Park, Philippine Racing Club Inc., Naic 4110, Philippines; 6 Regional Animal Disease Diagnostic Laboratory, Department of Agriculture Regional Field Office IV-A, Marawoy, Lipa City 4217, Philippines

**Keywords:** *Babesia*, *Theileria*, *Anaplasma*, goat, Philippines, tick-borne, PCR

## Abstract

Goats are key livestock animals and goat raising is an income-generating venture for smallholder farmers, supporting agricultural development in many parts of the world. However, goat production is often limited by various factors, such as tick-borne diseases. Goat piroplasmosis is a disease caused by apicomplexan parasites *Babesia* spp. and *Theileria* spp., while anaplasmosis is caused by bacterial *Anaplasma* spp. In the Philippines, the presence of *Babesia*, *Theileria*, and *Anaplasma* has not been reported in goats. In this study, DNA obtained from goats were molecularly screened for *Babesia/Theileria* and *Anaplasma*. Of 396, 77.02% (305/396) and 38.64% (153/396) were positive for piroplasma and *Anaplasma* using PCR assays targeting the 18S rRNA and 16S rRNA genes, respectively. Similarly, *Babesia ovis* was detected in six samples (1.52%). Representative *Babesia/Theileria* sequences shared 89.97–97.74% identity with each other and were most closely related to *T. orientalis*, *T. annulata*, and *Theileria* spp. Meanwhile, *Anaplasma* 16SrRNA sequences were related to *A. odocoilei*, *A. platys,* and *A. phagocytophilum*. This is the first molecular identification of *B. ovis, Theileria* spp., and *Anaplasma* spp. in goats from the Philippines.

## 1. Introduction

Babesiosis, theileriosis, and anaplasmosis are caused by tick-borne blood parasites of the genera *Babesia*, *Theileria,* and *Anaplasma*. These tick-borne diseases (TBDs) adversely affect livestock through direct and indirect losses in production. In small ruminants, the impact of TBDs has burdened farmers with losses linked to mortalities, less meat, milk, and wool produce, and increased costs for herd health management [1,2]. However, in endemic countries, TBDs are often overlooked despite being widespread in small ruminants, due to the lack of severe clinical manifestations during infection and strong tolerance through acquired natural immunity of infected hosts [3].

Several *Babesia* species can infect small ruminants, but the disease that develops varies between goats and sheep. *Babesia ovis* is fatal in sheep [4], while other species may have milder (*B. motasi*) [5] or low (*B. crassa*) [6] virulence. In goats, *B. ovis* causes subclinical infection [7,8], and *B. motasi* infects goats more frequently than sheep [5]. Additionally, some newly reported species have been identified in particular locations, namely *Babesia* sp. Xinjiang and *B. motasi*-like in China [9,10] and *Babesia* sp. in Turkey [11]. The clinical manifestations of babesiosis in small ruminants may include fever, anemia, jaundice, depression, and hemoglobinuria. In addition death may occur in severely affected animals [8]. Ticks of the genus *Rhipicephalus*, *Hyalomma*, and *Haemaphysalis* can transmit *Babesia* to small ruminants [12].

Theileriosis in small ruminants is caused by various species of *Theileria*, of which pathogenic species include *T. lestoquardi*, *T. luwenshuni* (*Theileria* sp. 1), and *T. uilenbergi* (*Theileria* sp. 2) [13]. Other *Theileria* species that can infect small ruminants are nonpathogenic [14], albeit considerably impact animal production [7]. The clinical disease in small ruminants may be accompanied by fever, lymph node swelling, icterus, hemorrhage, and diarrhea, while anemia, wasting, lack of appetite, and intermittent fever are observed during chronic infection [7]. Similar to babesiosis, infection in goats is less severe [5].

*Anaplasma* is the causative agent of anaplasmosis and is distributed globally, infecting a broad range of hosts. Small ruminants can get infected with several species, including *A. marginale*, *A. ovis*, *A. phagocytophilum*, and the newly discovered emerging pathogen *A. capra* [3,15,16]. Infected animals may experience fever, hemolytic anemia, loss of appetite, weight loss, and fatigue, which translate to reduced milk production in dairy small ruminants [3]. Biological vectors of *Anaplasma* are ixodid ticks, but mechanical vectors are also involved in the transmission, especially in places where the tick vectors are absent or rarely present [17].

Small ruminant production is an essential agro-socioeconomic activity that sustains agricultural development in many parts of the world by providing meat, milk, skin, and wool. Compared to other livestock, goat and sheep raising is attractive in rural households because of the relatively smaller resources and effort required to maintain them [18]. They can subsist on unpalatable low-quality fodder and browse and still be prolific, owing to the early sexual maturity, brief gestation duration, and short birth intervals [19]. In the Philippines, goat raising is a pillar of the mixed (crop–livestock) farming systems and provides supplemental income to smallholder farming families [20]. In 2021, there were 3.2 million heads of goats and the annual national production value was estimated at USD250 million [21]. Despite the huge contribution, goat production is suboptimal, and their full potential is not realized [18,22]. Several factors constrain caprine production in the Philippines, one of which is the high prevalence of parasites from genera *Eimeria* spp., *Fasciola* spp., *Haemonchus* spp., and *Trichostrongylus* spp. [20]. In contrast, the tick-borne parasites *Babesia*, *Theileria,* and *Anaplasma* have not been molecularly detected [23]. Therefore, in this study, we aimed to detect the molecular presence of these tick-borne pathogens in goats and determine the animal parameters associated with the detection.

## 2. Results

### 2.1. Sample Composition and Background Information

**Figure 1:**
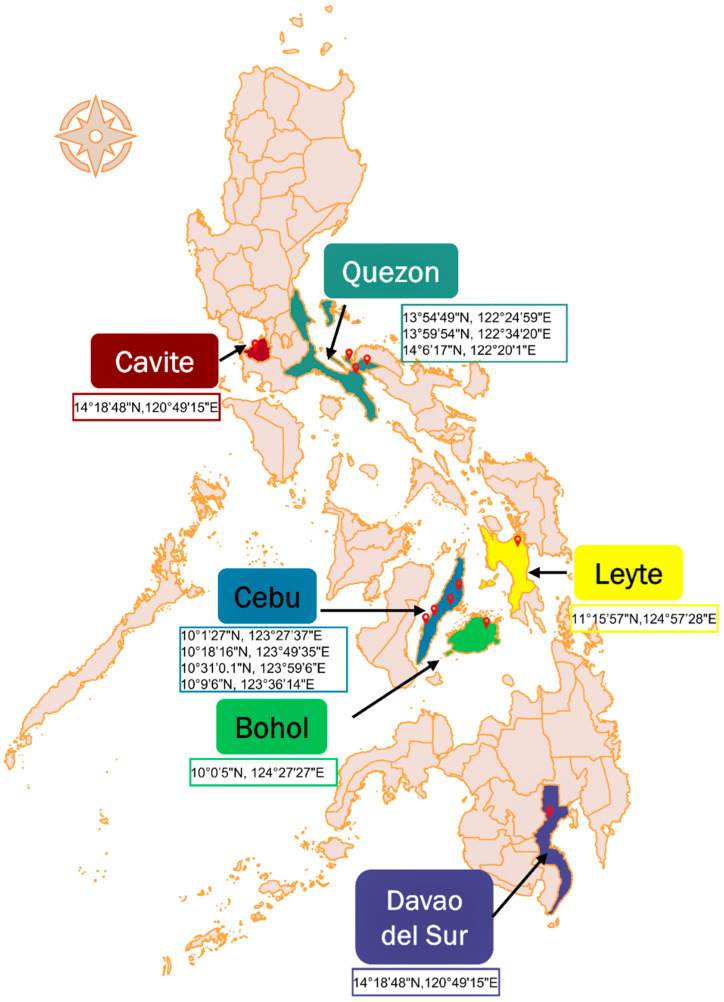
The Philippine map showing the six provinces (colored) where samples were collected from, with pinned sampling sites and GPS coordinates. The map was generated using the QGIS software [24].

In this study, samples were collected from 396 randomly selected goats across six provinces in the Philippines (Figure 1), namely, Cavite (n = 42), Quezon (n = 20), Bohol (n = 35), Cebu (n = 74), Leyte (n = 26), and Davao del Sur (n = 199) (Table 1). The goat population was comprised of the following: 60% adult (n = 237) and 40% young (n = 159); 87% female (n = 344) and 13% male (n = 52); 56% purebred (n = 222), 22% crossbred (n = 87), 15.4% Philippine native (n = 61), and unknown breed 6.6% (n = 26). The goats were raised in backyards by smallholder farmers, except those from Cebu and Bohol, which were domesticated in semicommercial and stock farms, respectively. The backyard goats were tethered and/or freely grazed, while goats from semicommercial farms were reared in semi-intensive and intensive systems.

### 2.2. Detection of Pathogens and Its Association with Host Parameters

Using the nested PCR assay targeting the 18S rRNA V4 hypervariable region of *Babesia/Theileria*, piroplasma DNA was detected in 305 (77.02%) samples (Table 1). The highest detection rates were recorded from one year or older adult (191/237; 80.59%), female (273/344; 79.36%), and purebred (179/222; 80.63) goats. Notably, piroplasma were most frequently detected in samples from Davao del Sur (183/199; 91.96%). Statistical analysis indicated that sex (*p* = 0.007), breed (*p* = 0.027), and location (*p* < 0.001) were associated with testing positive for piroplasma (Table 1). On the other hand, 153/396 (38.64%) samples were positive for *Anaplasma* spp. (*A. phagocytophilum*). *Anaplasma* detection rates were higher in young (77/159; 48.43%), male (25/52; 48.08%), and purebred (101/222; 45.50%) goats, with those from Quezon (16/20; 80.00%) showing the highest positivity rate (Table 1). Significant factors associated with *Anaplasma* positivity were age-group (*p* = 0.001), location (*p* < 0.001), and breed (*p* = 0.026). In addition, six (6/396; 1.52%) samples from Leyte (n = 3) and Cavite (n = 3) showed amplicons corresponding to the target of the *B. ovis* PCR assay.

### 2.3. Sequencing and Phylogenetic Analysis of Representative Sequences

**Figure 2:**
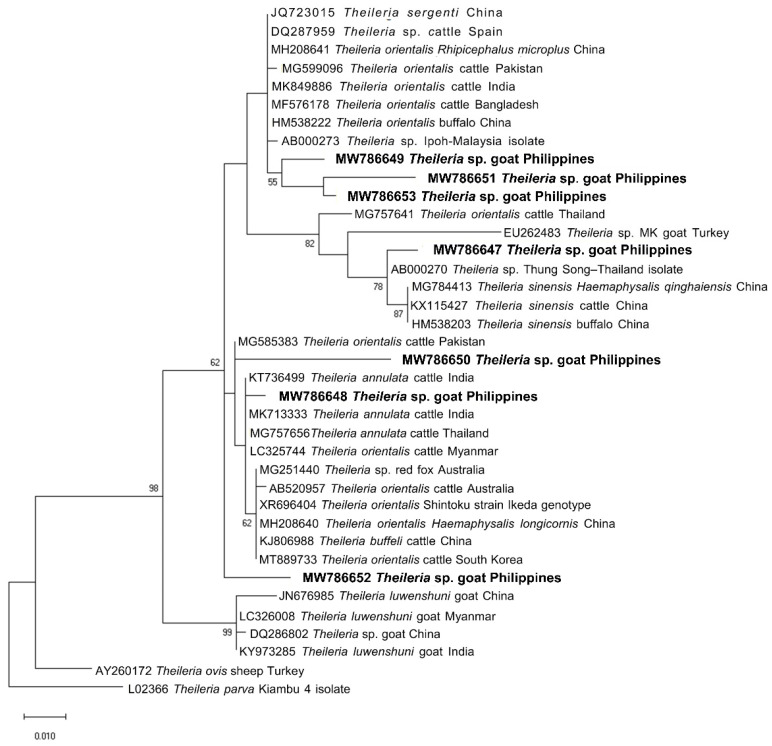
Phylogenetic analysis of piroplasma sequences obtained in this study (MW786647–MW786653) based on the 18S rRNA gene. The maximum likelihood tree was constructed using the Tamura-3 model plus discrete gamma distribution (+G, parameter = 0.3348). The phylogeny test was performed using the bootstrap analysis with 1000 iterations. The sequences obtained from the current study are shown in bold. *Theileria parva* was designated the outgroup.

**Figure 3:**
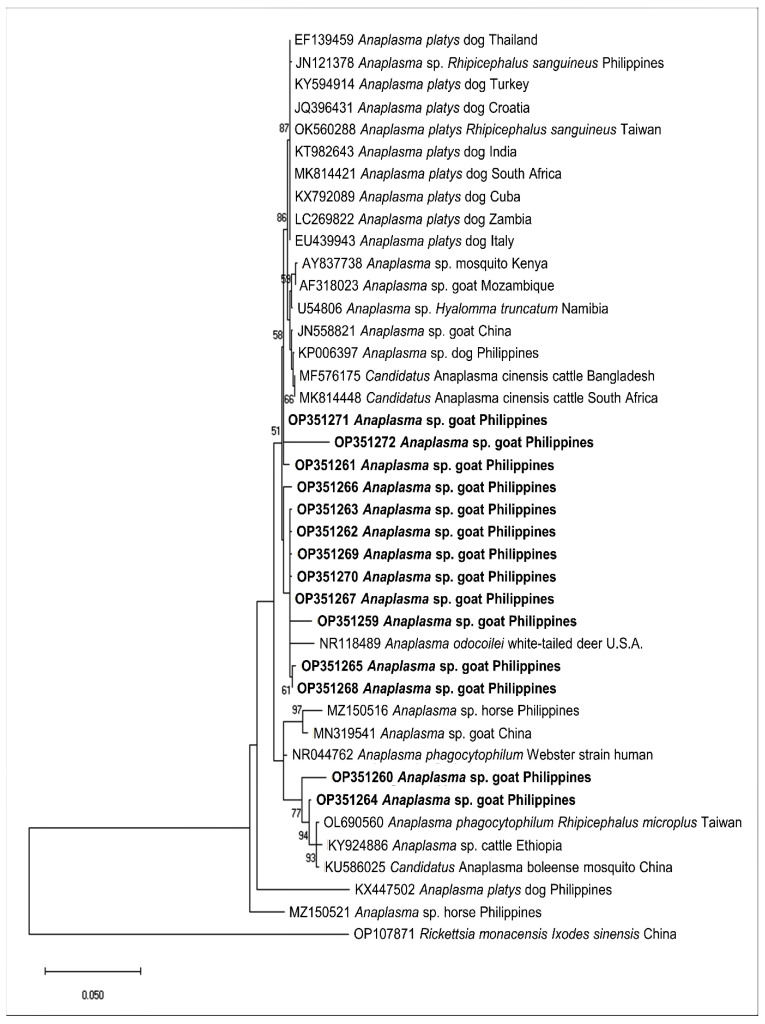
Phylogenetic analysis of *Anaplasma* spp. sequences obtained in this study (OP351259–OP351272) based on the 16S rRNA gene. The maximum likelihood tree was constructed using the Hasegawa-Kishino-Yano model plus discrete gamma distribution (+G, parameter = 0.3915). The phylogeny test was performed using the bootstrap analysis with 1000 iterations. The sequences obtained from the current study are shown in bold. *Rickettsia monacensis* was designated the outgroup.

**Figure 4:**
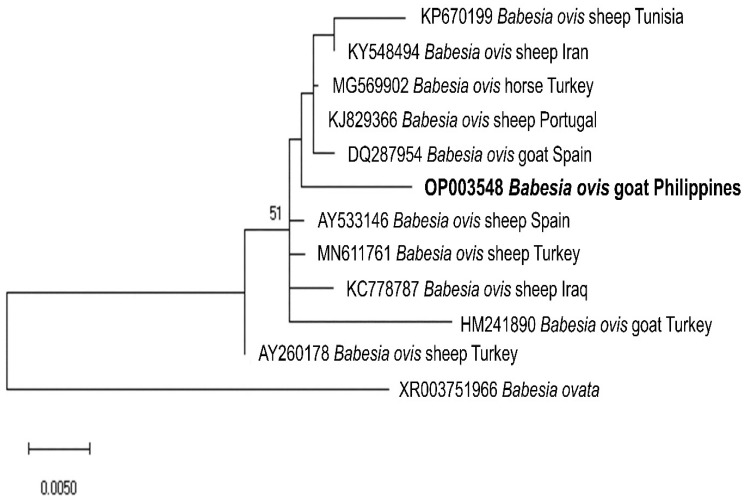
Phylogenetic analysis of the *B. ovis* sequence obtained in this study (OP003548) based on the ssu rRNA gene. The maximum likelihood tree was constructed using the Jukes–Cantor model with uniform rates among sites. The phylogeny test was performed using the bootstrap analysis with 1000 iterations. The sequence obtained from the current study is shown in bold. *Babesia ovata* was designated the outgroup.

We sequenced representative samples that showed strong bands for piroplasma (n = 7), *Anaplasma* sp. (n = 14), and *B. ovis* (n = 1) to determine their sequence identities and to analyze their phylogenetic relationships with previously published sequences in the GenBank sequence database. The seven sequences (MW786647–MW786653) were confirmed as *Theileria* species and exhibited intersequence identities of 89.97–97.74%. As shown in Figure 2, three isolates (MW786649; MW786651; MW786653) were located in a subclade with other *T. orientalis* isolates from China, Pakistan, India, Bangladesh, and Malaysia. One *Theileria* sp. (MW786653) was most closely related to *Theileria* sp. Thung Song isolate from Thailand (99.30% identity) and formed a sister clade with the Chinese *T. sinensis* isolates, while MW786648 shared 99.53% identity with *T. annulata* isolates from India and Thailand (Figure 2). MW786650 was similar to cattle isolate of *T. orientalis* from Pakistan, whereas MW786652 was located in a branch solitarily (Figure 2). The *Anaplasma* sp. sequences (OP351259–OP351272) obtained in this study shared the following identities with each other: 99.68% (OP351261 and OP351271); 98.81–99.89% (OP351262, OP351263, OP351265–OP351270); 98.38% (OP351260 and OP351264), 98.48% (OP351259 and OP351267); 97.84% (OP351271 and OP351272). Based on the *Anaplasma* 16S rRNA phylogenetic tree, OP351260 and OP351264 clustered with *A. phagocytophilum* in *Rhipicephalus microplus* from Taiwan, *Anaplasma* sp. in cattle from Ethiopia, and *Candidatus* A. boleense in mosquitoes from China, while OP351262, OP351263, and OP351265–OP351270 grouped together and were related to *A. odocoilei* from the US (Figure 3). In addition, OP351261, OP351271, and OP351272 were phylogenetically related to various *A. platys* isolates. The *B. ovis* isolate from the current study (OP003548) was closely related and had 99.82% identity with sheep, goat, and horse *B. ovis* isolates from Turkey, Iran, Spain, and Portugal (Figure 4).

## 3. Discussion

Herein, we present the first molecular identification of *Babesia, Theileria,* and *Anaplasma* in goats from the Philippines. A high detection rate of piroplasma DNA was recorded in goats (77.02%), which was higher than caprine *Babesia* and *Theileria* rates recorded in Pakistan (5–40.80%) [25,26], Turkey (21.40%) [27], Italy (11.70%) [3], China (11.90–34.70%) [28,29], Ethiopia (1.90%) [30], and Tunisia (4.70%) [31], while comparable to that from Malawi (72.70%) [32]. The detection rate (38.64%) of *Anaplasma* spp. in the current study was higher than what was observed in goats from Bangladesh (15.75%) [33], Thailand (13.50%) [34], Pakistan (7.80%) [35], and South Korea (7.62%) [36], but lower than in goats from China (58.50%) [37]. The relatively high detection rates may be due to several factors related to the climate, environment, host susceptibility, vector population density, and management production systems [3,27,38]. In addition, *B. ovis* was detected in 1.52% of the goat samples and was present in two provinces (Leyte and Cavite). The current non-detection of *B. ovis* in Cebu goats agrees with the results of a previous molecular investigation where *Babesia* was not detected in caprine blood samples [23].

Significant association between host parameters, including sex, age-group, breed, and location, and pathogen detection was noted in the present study. Piroplasma positivity in female goats was significantly higher than in male ones, while studies on small ruminants in Ethiopia [30], Turkey [39], and Tunisia [40] found goat sex to be negligible. On the other hand, *Anaplasma* spp. detection in young goats was significantly higher than in adults, which is parallel to the findings in Pakistan goats [35]. Moreover, detection of *Anaplasma* spp. and *Babesia/Theileria* were significantly associated with goat breed, wherein a higher number of purebred or exotic goats tested positive compared to upgraded and native goats. Earlier surveys observed a similar trend, where the indigenous goat breeds were described to have higher resistance to piroplasma [40,41] and *Anaplasma* [42]. Information available on goat breed resistance to TBDs is scarce, but one possible explanation may be the greater susceptibility of exotic goat breeds to tick bites compared to local breeds [43]. Piroplasma and *Anaplasma* detection rates significantly differed among the location of the goats. The same was observed in goat herds from China [29], Tunisia [40], and Oman [44]. Location-specific factors in this study, such as management practices and macroclimatic conditions that affect tick proliferation, may be attributed to the different sampling sites characterized by diverse topography, terrain, microclimate, and fauna.

Sequencing and phylogenetic analysis confirmed that the representative *Theileria* sp. sequences obtained in this study were most closely related to *T. orientalis*, *T. annulata*, and *Theileria* sp. Thung Song isolate. Four *Theileria* isolates in the present study (MW786649; MW786651; MW786653) were similar to *T. orientalis* isolates from other locations. Members of the *T. orientalis* complex have been reported in cattle from the Philippines in previous studies [45,46,47]. More notably, we obtained an isolate highly similar to *T. annulata*. *T. annulata* is a species that can infect goats and causes a potentially fatal disease in cattle [48]. However, it should be confirmed whether the infected goats can be inapparent carriers of various *T. orientalis* genotypes and *T. annulata* and if they are able to spread the pathogens to bovids, as in sheep [48,49,50]. *Theileria* sp. Thung Song is an isolate from dairy cattle in Thailand and was genotypically divergent from other benign *T. orientalis* types (*T. buffeli* and *T. sergenti*) [51,52]. Its detection in goats in the current study may indicate host shifting of this parasite. We also obtained an isolate phylogenetically distinct from other analyzed sequences (MW786652; 98.75% identity with *Theileria* sequences in GenBank), which may be a new *Theileria* sp., although more studies are needed to verify this claim. On the other hand, *T. luwenshuni,* which is a common species in goats reported from nearby Southeast Asian countries, namely, Thailand [34,53,54,55], Myanmar [56], and Vietnam [50,57,58], was not confirmed in the obtained sequences.

In this study, *Anaplasma* isolates (OP351259–OP351272) closely related to *A. odocoilei, A. phagocytophilum*, and *A. platys* were confirmed in goats from the Philippines. *A. odocoilei* is a species causing chronic *Anaplasma* infection in white-tailed deer discovered in the US [59]. *A. odocoilei* does not cause severe clinical disease in experimentally infected white-tailed deer, and natural infections have only been detected from North America and South America [60,61,62]. Additionally, we obtained isolates (OP351261 and OP351271) highly similar to *A. platys* (99.10% and 99.46% identity with other *A. platys* isolates, respectively) and OP351272, a potential novel *A. platys*-like isolate (97.30% highest identity with GenBank *A. platys* isolates). A novel *A. platys*-like species that can be vertically transmitted to the goat’s offspring has been identified in China recently [63]. Furthermore, a previous study indicated that a couple of novel *A. phagocytophilum*-like and *A. ovis*-like variants are circulating in Philippine horses [64]. Thus, the isolates obtained from the current study warrant further probing on their genetic characteristics and clinical impact of the infections they inflict on goats.

Despite the high rate of positivity, clinical signs associated with TBDs were not observed. This may indicate the endemicity of these pathogens in goats in the Philippines. Likewise, this may correspond to persistent infections, a characteristic of natural infections in places where the disease is presumed endemic [7]. While *B. ovis* causes acute and severe disease in sheep, natural infection with *B. ovis* in goats is rarely clinical [8]. This was evident in the positive goats in the current study. In the case of *Theileria*- and *Anaplasma*-positive goats, the impact of subclinical infections should not be ignored because the pathogenicity of different species and genotypes vary depending on the host [15,65].

Some aspects were outside the scope of this study. For instance, few samples were subjected to sequencing analysis and species confirmation was performed by partial amplification of one fragment from one gene. Therefore, additional studies based on species-specific detection should be conducted to elucidate the species diversity of *Babesia*, *Theileria,* and *Anaplasma* in goats from the Philippines. In this study, the tick vectors were not determined. Since tethering and freely grazing systems are the more common production system in the Philippines [20], there are more opportunities for the ticks to feed on the host as goats are exposed to vegetation where questing ticks are abundant. The most likely vector of piroplasmas and *Anaplasma* is the ubiquitous *R. microplus* ticks, which was found infesting goats in Bulacan province [66]. The tick species *R. microplus* and *Haemaphysalis bispinosa* infesting goats from neighboring countries Thailand [34,67] and Malaysia [68] were also confirmed to be carrying *T. luwenshuni*. Similarly, *R. sanguineus* sensu lato (s.l.) ticks, the vector of canine *A. platys* in the Philippines [69,70], may also be implicated as the vector of *A. platys*-like variants detected in the present study. The three-host tick *R. sanguineus* s.l. commonly infests dogs, its main host, but humans and other animals may also be incidentally infested [71]. A majority of goats in this study were raised in backyards and regularly interacted with companion animals (dog and cats), which may have possibly exposed goats to the former’s tick vectors. Moreover, the possibility of other transmission means, particularly, the role of mechanical transmission by insect vectors other than ticks, such as blood-sucking arthropods *Tabanus, Stomoxys,* and mosquitoes [17], should be further investigated.

## 4. Materials and Methods

### 4.1. Ethics Statements

Field sampling and animal handling protocols were conducted in accordance with the Philippine Animal Welfare Act (Republic Act 10631) and the guidelines set by the Institutional Animal Care and Use Committee of the University of the Philippines Cebu and Cavite State University. Experimental procedures and methodologies related to this study were permitted by Obihiro University of Agriculture and Veterinary Medicine, Obihiro, Hokkaido, Japan (permits 20–128 and 1723–4). The farmers and owners of the animals were oriented regarding the purpose of the study and provided verbal consent prior to the start of the sample collection.

### 4.2. Sample Collection and Sampling Sites

In this study, 396 whole-blood samples from randomly chosen goats were collected. The goats were randomly chosen irrespective of sex, age, and breed from March 2017 to March 2020. Sampling was done in backyards and farms selected by convenience in the provinces of Cavite (n = 42), Quezon (n = 20), Bohol (n = 35), Cebu (n = 74), Leyte (n = 26), and Davao del Sur (n = 199), Philippines. The specific sampling sites and their GPS coordinates are shown in Figure 1. Approximately 2 mL of blood was collected via venipuncture of the jugular vein of the goats into sterile EDTA tubes and kept cool until processing in the laboratory.

### 4.3. Genomic DNA Isolation

Genomic DNA was extracted using the QIAamp^®^ DNA Blood Mini Kit (Qiagen, Hilden, Germany) according to the manufacturer’s instructions. About 200 μL of whole blood was used for the DNA extraction using the column-based blood kit. The DNA samples were transported to the National Research Center for Protozoan Diseases, Obihiro, Hokkaido, Japan and stored at −30 °C until use. Quality and the estimated concentration of the extracted DNA samples were checked using the NanoDrop™ 2000 spectrophotometer (Thermo Fisher Scientific, Waltham, MA, USA) prior to screening the samples.

### 4.4. PCR Assays for Pathogen Detection

The PCR conditions performed in this study are referred to in Table 2. The samples were processed using a nested PCR assay targeting the hypervariable V4 region of the 18S rRNA gene of piroplasma [72,73] and 16S rRNA of *Anaplasma* spp. (*A. phagocytophilum*) [74]. In addition, a single specific primer set amplifying the *B. ovis* 18S rRNA gene was also used [75]. For the nested PCR assays, both first and final reactions were run to a final volume of 10 µL consisting of 1× ThermoPol^®^ buffer (New England Biolabs, Ipswich, MA, USA), 2 mM of dNTP mix (New England Biolabs) 2 µM of forward and reverse primers, 0.25 U of Taq DNA polymerase (New England Biolabs), and 2 µL of genomic DNA sample for the first assay or 1 µL of the PCR product for the nested assay. For the screening of *B. ovis*, the conventional assay was performed similarly to the aforementioned setup, except for the final concentration of primers (5 µM). The company-provided thermocycling conditions were followed, with the annealing temperature for each assay listed in Table 2. Positive (DNA samples confirmed positive for *Theileria* sp., *B. ovis*, and *Anaplasma* sp. [76]) and negative controls (UltraPure™ DNase/RNase-Free distilled water; Invitrogen, Waltham, MA, USA) were run alongside the samples in each assay. Visualization of amplicons after exposure to UV light was done after electrophoresis of PCR products in 1.5% agarose gel and staining with ethidium bromide solution.

### 4.5. Sequencing and Phylogenetic Analysis

Amplicons were purified using NucleoSpin^®^ Gel and PCR Clean-up kit (Macherey Nagel, Düren, Germany). Then, the purified amplicons were cloned by ligation into pGEM^®^-T Easy Vector (Promega Corporation, Madison, WI, USA) and transformation in *Escherichia coli* DH5α strain calcium-competent cells. After overnight incubation of positive transformants, high-density bacterial cultures were lysed and plasmids were purified using NucleoSpin Plasmid QuickPure Kit (Macherey Nagel). Purified plasmids were sequenced by the Sanger sequencing method using BigDye^™^ Terminator v3.1 Cycle Sequencing Kit (Applied Biosystems, Foster City, CA, USA) and ABI Prism 3100 Genetic Analyzer (Applied Biosystems).

Forward and reverse reads were manually trimmed and overlapped to assemble the sequences. Shared identities between presently obtained and previously deposited sequences were determined by NCBI BLASTn search, while the identity matrix generated from EMBL Clustal Omega multiple sequence alignment [77] determined the intersequence percentage identities. After nucleotide alignment by Clustal W and determination of the best DNA model, the maximum likelihood trees were constructed by phylogeny testing using the bootstrap method with 1000 replications. All analyses related to phylogeny were conducted using the Molecular Evolutionary Genetics Analysis (MEGA) X software [78]. The sequences obtained from this study were banked in the NCBI GenBank with accession numbers MW786647–MW786653 for *Theileria* sp. 18S rRNA (372–426 bp), OP003548 for *B. ovis* 18S rRNA (552 bp), and OP351259–OP351272 for *Anaplasma* sp. 16S rRNA (923–925 bp).

### 4.6. Statistical Analyses

Association between *Babesia/Theileria* sp. and *Anaplasma* sp. positivity (dependent variable) and animal parameters (categorical independent variables), namely, sex, age group, breed, and location, was evaluated. Background data of animal samples were available for all except for the breeds of goats from Leyte; thus, we excluded them in the analysis for the breed variable. Fisher’s exact test was used to calculate the exact *p* values, whereas if not applicable, Pearson’s chi-square test was employed to calculate the approximate *p* values. A *p* value of <0.05 was considered significant. All statistical analyses were performed using GraphPad Prism 8 (GraphPad software, San Diego, CA, USA).

## Figures and Tables

**Table 1:** Molecular detection of *Babesia/Theileria* spp. and *Anaplasma* spp. based on sex, age-group, location, and breed of goats.

Variable	N	*Babesia/Theileria* spp.		*Anaplasma* spp.	
		No. of Positives (%)	*p* Value	No. of Positives (%)	*p* Value
Age-group					
Young (<1 year)	159	114 (71.70)	0.051	77 (48.43)	0.001 **
Adult (≥1 year)	237	191 (80.59)		76 (32.07)	
Sex					
Male	52	32 (61.54)	0.007 **	25 (48.08)	0.169
Female	344	273 (79.36)		128 (37.21)	
Location					
Davao del Sur	199	183 (91.96)	<0.001 ***	82 (41.21)	<0.001 ***
Cebu	74	35 (47.30)		37 (50.00)	
Bohol	35	16 (45.71)		10 (28.57)	
Quezon	20	8 (40.00)		16 (80.00)	
Leyte	26	24 (92.31)		2 (7.69)	
Cavite	42	39 (92.86		6 (14.29)	
Breed ^#^					
Purebred (Anglo-Nubian or Boer)	222	179 (80.63)	0.027 *	101 (45.50)	0.026 *
Crossbred or upgrades	87	58 (66.67)		25 (28.74)	
Philippine native	61	44 (72.13)		25 (40.98)	
Total	396	305 (77.02)		153 (38.64)	

Asterisks indicate significant differences: * <0.05; ** <0.01; *** <0.001. ^#^ Goats of unknown breeds (n = 26) were excluded from the analysis.

**Table 2:** List of PCR primers and conditions used in the study.

Pathogen	Target Gene	Primer Sequence	Annealing Temperature (°C)	Target Length (bp)	Reference
Piroplasma (*Babesia*/*Theileria*)	18S rRNA (V4 hypervariable region)	F1: 5’—GAGGTAGTGACAAGAAATAACAATA—3’	50	~460–520	[72]
		R1: 5’—TCTTCGATCCCCTAACTTTC—3’			
		F2: 5’—GACACAGGGAGGTAGTGACAAG—3’	60	~390–420	[73]
		R2: 5’—CTAAGAATTTCACCTCTGACAGT—3’			
*Babesia ovis*	Small subunit 18S rRNA	F: 5’—TGGGCAGGACCTTGGTTCTTCT—3’	62	~549	[75]
		R: 5’—CCGCGTAGCGCCGGCTAAATA—3’			
*Anaplasma* spp. (*A. phagocytophilum*)	16S rRNA	F1: 5’—TCCTGGCTCAGAACGAACGCTGGCGGC—3’	50	~1433	[74]
		R1: 5’—AGTCACTGACCCAACCTTAAATGGCTG—3’			
		F2: 5’—GTCGAACGGATTATTCTTTATAGCTTGC—3’	50	~925	
		R2: 5’—CCCTTCCGTTAAGAAGGATCTAATCTCC—3’			

## Data Availability

The datasets generated during and/or analyzed during the current study are available from the corresponding author on reasonable request.
